# Ceftiofur Resistance in *Salmonella enterica* Serovar Heidelberg from Chicken Meat and Humans, Canada

**DOI:** 10.3201/eid1601.090729

**Published:** 2010-01

**Authors:** Lucie Dutil, Rebecca Irwin, Rita Finley, Lai King Ng, Brent Avery, Patrick Boerlin, Anne-Marie Bourgault, Linda Cole, Danielle Daignault, Andrea Desruisseau, Walter Demczuk, Linda Hoang, Greg B. Horsman, Johanne Ismail, Frances Jamieson, Anne Maki, Ana Pacagnella, Dylan R. Pillai

**Affiliations:** Public Health Agency of Canada, Saint-Hyacinthe, Québec, Canada (L. Dutil, D. Daignault); Public Health Agency of Canada, Guelph, Ontario, Canada (R. Irwin, R. Finley, B. Avery, L. Cole, A. Desruisseau); Public Health Agency of Canada, Winnipeg, Manitoba, Canada (L.K. Ng, W. Demczuk); Ontario Veterinary College, Guelph (P. Boerlin); Institut National de Santé Publique du Québec, Sainte-Anne-de-Bellevue, Québec, Canada (A.-M. Bourgault, J. Ismail); British Columbia Centre for Disease Control, Vancouver, British Columbia, Canada (L. Hoang, A. Pacagnella); Saskatchewan Disease Control Laboratory, Regina, Saskatchewan, Canada (G. Horsman); Ontario Agency for Health Protection and Promotion, Toronto, Ontario, Canada (F. Jamieson, A. Maki, D.R. Pillai)

**Keywords:** Salmonella enterica serovar Heidelberg, Escherichia coli, antimicrobial resistance, humans, chickens, Canada, bacteria, expedited, research

## Abstract

The Canadian Integrated Program for Antimicrobial Resistance Surveillance describes a strong correlation (r = 0.9, p<0.0001) between ceftiofur-resistant *Salmonella enterica* serovar Heidelberg isolated from retail chicken and incidence of ceftiofur-resistant *Salmonella* serovar Heidelberg infections in humans across Canada. In Québec, changes of ceftiofur resistance in chicken *Salmonella* Heidelberg and *Escherichia coli* isolates appear related to changing levels of ceftiofur use in hatcheries during the study period, from highest to lowest levels before and after a voluntary withdrawal, to increasing levels after reintroduction of use (62% to 7% to 20%, and 34% to 6% to 19%, respectively). These events provide evidence that ceftiofur use in chickens results in extended-spectrum cephalosporin resistance in bacteria from chicken and humans. To ensure the continued effectiveness of extended-spectrum cephalosporins for treating serious infections in humans, multidisciplinary efforts are needed to scrutinize and, where appropriate, limit use of ceftiofur in chicken production in Canada.

## Abstract 2

Use of this drug in chickens may limit effectiveness of cephalosporins in treating human infections.

*Salmonella enterica* serovar Heidelberg ranks among the top 3 serovars isolated from persons infected with *Salmonella* in Canada (*1*). It is more frequently reported in North America than in other regions of the world (*2*). Although many *Salmonella* Heidelberg infections result in mild to moderate illness, the bacterium also causes severe illness with complications such as septicemia, myocarditis, extraintestinal infections, and death (3,*4*). *Salmonella* Heidelberg appears more invasive than other gastroenteritis-causing serovars; ≈9% of isolates of this serovar received through the Canadian Integrated Program for Antimicrobial Resistance Surveillance (CIPARS) during 2003–2005 were recovered from blood samples (*5*). Treatment with antimicrobial agents may be life-saving in the case of invasive infections.

Sources of human *Salmonella* Heidelberg infection include consumption of poultry or eggs and egg-containing products (*6*–*10*). In Canada, *Salmonella* Heidelberg is commonly isolated from healthy chickens from farm, abattoir, and retail sources (*11*,*12*). It also has been isolated, although less frequently, from ground beef, pork, and turkey meat (*13*–*15*) and from clinical samples from various animal species (*12*).

Ceftiofur is an extended-spectrum cephalosporin drug approved in Canada for use with numerous label indications in cattle, swine, horses, sheep, turkeys, dogs, and cats. Ceftiofur is also injected in ovo to control *Escherichia coli* omphalitis in broiler chickens; this use is not an approved label indication.

A major public health concern is that use of third-generation cephalosporins, such as ceftiofur, in food animals is leading to resistance to other extended-spectrum cephalosporins, such as ceftriaxone and cephamycins (*16*–*20*), a group of antimicrobial agents used to treat a wide variety of human infections. Among other indications, ceftriaxone is the drug of choice for treating severe or invasive salmonellosis in children and pregnant women (*16*,*17*) where fluoroquinolones are not approved and treatment options are limited. Accordingly, third-generation cephalosporins have been classified as Critically Important Antimicrobials in Human Medicine by the World Health Organization (*21*) and as Class 1 Very High Importance in Human Medicine by the Canadian Veterinary Drugs Directorate, Health Canada (*22*).

In Canada, ceftiofur resistance in bacteria from healthy animals or food is mainly reported in chicken *Salmonella* Heidelberg isolates originating from farm, abattoir, and retail samples and in chicken abattoir and retail generic *E. coli* isolates (*11*,*12*). It also is occasionally reported in *Salmonella* isolates from sick animals or in bovine and porcine abattoir or retail *E. coli* isolates but at much lower frequency (*12*).

The objective of this study is to highlight the correlation between ceftiofur-resistant *Salmonella* Heidelberg isolated from retail chicken and the incidence of ceftiofur-resistant *Salmonella* Heidelberg infections in humans across Canada. Public health concerns raised by publication of the CIPARS 2003 annual report, specifically the higher rates of ceftiofur resistance in *Salmonella* Heidelberg isolates from chicken meat than from humans, prompted Québec broiler chicken hatcheries to voluntarily interrupt the extralabel in ovo use of ceftiofur during 2005–2006 (*23*). This study therefore also describes variations in ceftiofur resistance among chicken and human *Salmonella* Heidelberg and chicken *E. coli* strains in that province before, during, and after the voluntary withdrawal.

## Materials and Methods

CIPARS is a national program led by the Public Health Agency of Canada (PHAC) dedicated to the preservation of effective antimicrobial drugs for humans and animals through the collection, integration, analysis, and communication of trends in antimicrobial resistance in selected bacterial organisms. Data presented here were collected during 2003–2008 from CIPARS’ surveillance of human clinical *Salmonella* isolates and *E. coli* and *Salmonella* isolates from retail chicken. Detailed methods for sample collection, bacterial isolation, antimicrobial resistance testing, and data analysis are described in CIPARS’s reports (*12*).

### Sample Collection

#### Human *Salmonella* Isolates

Hospital-based and private clinical laboratories isolated and forwarded human *Salmonella* isolates to their Provincial Public Health Laboratory (PPHL). PPHLs forwarded *Salmonella* isolates to the Enteric Diseases Program, National Microbiology Laboratory (NML), PHAC, for phage type characterization and antimicrobial resistance testing. All isolates (outbreak and nonoutbreak) received passively by the Saskatchewan PPHL were forwarded; the more populated provinces (British Columbia, Ontario, and Québec) forwarded isolates received from days 1–15 of each month. Only 1 isolate per patient was kept for the analysis.

#### Retail Meat Samples

To use a similar geographic scale as CIPARS surveillance of human clinical *Salmonella* isolates and because we expected a certain level of provincial clustering in food distribution, we designed the study of CIPARS retail surveillance to provide a representative measurement of what consumers from each province were exposed to through ingestion of improperly cooked raw meat or cross-contamination. Randomization and weighted allocation of samples according to demography of the human population ensured that the data generated through retail sampling were representative and reliable at the provincial level. Retail raw chicken samples (most often chicken thigh with skin on) were collected as part of CIPARS retail program that purchases samples weekly (Ontario and Québec) or biweekly (Saskatchewan, British Columbia) from chain, independent, and butcher stores in 15–18 randomly selected census divisions in each participating province. Retail surveillance was initiated in Ontario and Québec in mid-2003 and at the beginning of 2005 in Saskatchewan. Surveillance also was conducted during part of 2007 and all of 2008 in British Columbia.

### Microbiologic Analysis

#### Recovery of Isolates from Retail Chicken Meat

Primary isolations of *E. coli* and *Salmonella* spp. were conducted at the Laboratory for Foodborne Zoonoses, PHAC. Every retail chicken meat sample received was cultivated for *Salmonella*, but only 1 of every 2 samples was systematically selected to be tested for generic *E. coli* isolation. Incubated peptone rinses of chicken meat samples were streaked on eosin-methylene blue agar (Becton Dickinson, Sparks, MD, USA). Presumptive *E. coli* colonies were identified by using the Simmons’ citrate and indole tests. Colonies showing negative indole results were identified by using the API 20E (bioMérieux Clinical Diagnostics, Marcy l’Étoile, France). All chicken samples were tested for *Salmonella* with a modified MFLP-75 method of the Compendium of Analytical Methods (*24*). Incubated peptone rinses were injected into modified semisolid Rappaport-Vassiliadis media. Presumptive *E. coli* colonies were injected into triple sugar iron and urea agar slants and subjected to the indole test. Presumptive *Salmonella* isolates were verified by slide agglutination using PolyA-I and Vi *Salmonella* antiserum (Difco, Becton Dickinson). *Salmonella* isolates were shipped between laboratories on a tryptic soy agar slant by priority courier. No selective media were used to isolate ceftiofur-resistant bacteria.

#### Serotyping, Phage Typing, and Susceptibility Testing

Human and chicken *Salmonella* isolates were serotyped and phage typed by using published methods (*25*–*28*). MICs were determined by the NML (human isolates) and the Laboratory for Foodborne Zoonoses, PHAC (chicken isolates) by the broth microdilution method (Sensititre Automated Microbiology System; Trek Diagnostic Systems Ltd., Westlake, OH, USA). *Salmonella* and *E. coli* isolates were tested by using the National Antimicrobial Resistance Monitoring System custom susceptibility plate for gram-negative bacteria. The breakpoint used to determine ceftiofur resistance was >4 μg/mL (*29*).

### Data Analysis

We analyzed data using SAS version 9.1 (SAS Institute Inc., Cary, NC, USA). The yearly proportion of retail chicken samples contaminated with ceftiofur-resistant *Salmonella* Heidelberg (or *E. coli*) and the incidence rate of human infection with ceftiofur-resistant *Salmonella* Heidelberg was calculated as described in CIPARS 2006 annual report (*12*). The Pearson product-moment correlation was used to verify the correlation between ceftiofur-resistant *Salmonella* Heidelberg isolated from retail chicken and human incidence estimates by using the Pearson option in the PROC CORR procedure in SAS. We computed the overall correlation coefficient using data across all provinces under study and computed a specific coefficient for provinces with >5 observations (*30*)

To describe ceftiofur resistance changes by quarter and reduce the noise around the estimate caused by the small number of observations per quarter, we computed a nonweighted rolling average of the prevalence of ceftiofur resistance using data from the current quarter and the previous 2 quarters for chicken *E. coli,* chicken *Salmonella* Heidelberg, and human *Salmonella* Heidelberg isolates from the province of Québec. We tested differences in ceftiofur resistance between years with SAS using χ^2^ or Fisher exact tests when appropriate.

## Results

### Ceftiofur-Resistant *Salmonella* Heidelberg Isolated from Retail Chickens and from Humans

**Figure 1:**
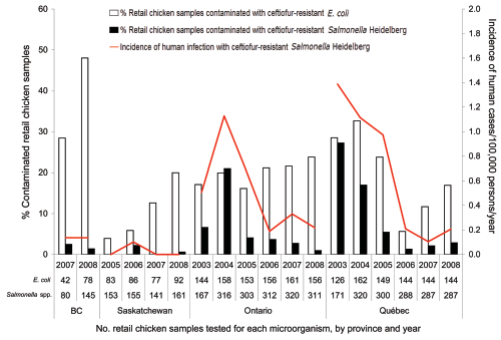
Prevalence of retail chicken contaminated with ceftiofur-resistant *Escherichia coli* and *Salmonella enterica* serovar Heidelberg and incidence of human infections from ceftiofur-resistant *Salmonella* Heidelberg in Canada.

Across Canada, the annual percentage of chicken samples contaminated with ceftiofur-resistant *Salmonella* Heidelberg correlated strongly with the annual incidence of human cases related to this type of isolate (r = 0.91, p<0.0001) (Figure 1). This strongly significant correlation held across time and within different Canadian provinces (Ontario, r = 0.93, p<0.01; Québec, r = 0.89, p = 0.01).

Changes in ceftiofur resistance alone did not explain a number of the temporal changes in exposure (*12*). For example, in Ontario, the decrease in the prevalence of retail chicken contaminated with ceftiofur-resistant *Salmonella* Heidelberg isolates during 2004–2008 (Figure 1) was linked to a decrease in ceftiofur resistance from 58% to 14% (Table) and a decrease in the prevalence of *Salmonella* Heidelberg in chicken from 61% to 15% of all *Salmonella* isolates. In Québec, the decrease in contamination of chicken with ceftiofur-resistant *Salmonella* Heidelberg strains from 2003 to 2004 (Figure 1) was related mainly to a decrease in the prevalence of *Salmonella* Heidelberg (from 71% to 48%) in chicken, whereas the decrease from 2004 to 2006 was attributable mainly to a drop in ceftiofur resistance from 62% to 7% (Table). In British Columbia, the low level of chicken contamination with ceftiofur-resistant *Salmonella* Heidelberg strains resulted mainly from the rarity of *Salmonella* Heidelberg (only 11% of all *Salmonella* in 2007–2008), and low exposure levels in Saskatchewan were related mainly to low ceftiofur resistance among *Salmonella* Heidelberg (Table).

**Table:** Prevalence of ceftiofur resistance among human and retail chicken *Salmonella* serovar Heidelberg isolates and retail chicken *Escherichia coli* isolates from Canadian provinces surveyed during 2003–2008

Isolate/province	Prevalence of ceftiofur resistance, % (no. resistant isolates/total no. isolates tested)					
	2003	2004	2005	2006	2007	2008
Human clinical *Salmonella* Heidelberg						
Québec	31 (52/167)	36 (42/116)	35 (37/106)	8 (8/96)	6 (4/63)	12 (8/65)
Ontario	18 (31/172)	38 (70/185)	30 (42/140)	10 (12/122)	22 (21/94)	32 (7/22)
Saskatchewan			0 (0/15)	7 (1/14)	0 (0/11)	0 (0/7)
British Columbia					23 (3/13)	19 (3/16)
Chicken retail *Salmonella* Heidelberg						
Québec	65 (13/20)	62 (18/29)	33 (4/12)	7 (1/14)	19 (6/32)	18 (7/38)
Ontario	16 (3/19)	58 (19/33)	27 (3/11)	21 (3/14)	21 (9/42)	14 (3/21)
Saskatchewan			0 (0/5)	13 (1/8)	0 (0/9)	8 (1/12)
British Columbia					50 (2/4)	67 (2/3)
Chicken retail *E. coli*						
Québec	32 (36/111)	34 (54/158)	25 (35/142)	6 (8/135)	13 (17/128)	18 (24/131)
Ontario	18 (24/136)	21 (32/150)	17 (25/145)	22 (34/152)	22 (35/157)	24 (36/150)
Saskatchewan			4 (3/82)	6 (5/85)	13 (10/75)	20 (18/92)
British Columbia					29 (12/42)	49 (34/70)

### Ceftiofur-Resistant *E. coli* Isolated from Retail Chicken

Retail chicken generally was more frequently contaminated with ceftiofur-resistant commensal *E. coli* than with ceftiofur-resistant *Salmonella* Heidelberg isolates (Figure 1). The proportion of chicken contaminated with ceftiofur-resistant *E. coli* (Figure 1) closely followed changes in ceftiofur resistance (Table) because commensal *E. coli* was recovered from almost all (89%–100%) chicken samples collected. Exposure to ceftiofur-resistant *E. coli* strains appeared to have increased in recent years in Canada (Figure 1). In 2008, exposure to ceftiofur-resistant *E. coli* strains was highest in British Columbia and lowest in Québec.

### Temporal Changes in Ceftiofur Resistance in the Province of Québec, 2003–2008

**Figure 2:**
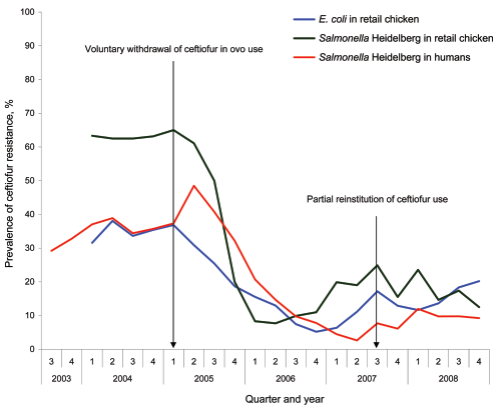
Prevalence of ceftiofur resistance (moving average of the current quarter and the previous 2 quarters) among retail chicken *Escherichia coli*, and retail chicken and human clinical *Salmonella enterica* serovar Heidelberg isolates during 2003–2008 in Québec, Canada.

In 2003–2004, >60% of the chicken *Salmonella* Heidelberg isolates were ceftiofur resistant, and ceftiofur resistance among chicken *E. coli* and human *Salmonella* Heidelberg isolates varied from 30% to 40% (Figure 2). Ceftiofur resistance declined sharply immediately after the first quarter of 2005 among chicken *E. coli* and *Salmonella* Heidelberg isolates, and a similar decline began in the next quarter among human *Salmonella* Heidelberg isolates (Figure 2). This decline steadily continued until the end of 2006. As a result, the prevalence of ceftiofur resistance significantly decreased from 2004 to 2006 among chicken (62% to 7%; p<0.001) and human (36% to 8%; p<0.0001) *Salmonella* Heidelberg isolates and chicken *E. coli* isolates (34% to 6%; p<0.0001 [Table]). Then, from 2006 to 2008, the prevalence of ceftiofur resistance significantly increased among chicken *E. coli* isolates (6% to 18%; p = 0.002), and prevalence of ceftiofur resistance increased, but not significantly, among *Salmonella* Heidelberg from chicken (7% to 18%; p = 0.32) and human (8% to 12%; p = 0.41) isolates (Table).

## Discussion

CIPARS data clearly indicate a temporal association between changing levels of contamination of retail chicken with ceftiofur-resistant *Salmonella* Heidelberg strains and incidence of ceftiofur-resistant *Salmonella* Heidelberg infection in humans. This correlation is strong and applies to different regions of Canada. Our observation is consistent with published results from outbreak investigations and case-control studies suggesting that chicken products are a source of human infection with *Salmonella* Heidelberg in Canada (*7*,*8*).

Although humans potentially can become infected with ceftiofur-resistant *Salmonella* Heidelberg from sources other than chicken, chicken appears the most likely source in Canada. Ceftiofur-resistant *Salmonella* Heidelberg has never been reported among CIPARS porcine *Salmonella* of abattoir origin, and it has not been detected among retail pork, abattoir beef, or retail beef, in which *Salmonella* prevalence remains <2% (*12*). Data generated by National Antimicrobial Resistance Monitoring System retail surveillance in the United States indicated that 17% of *Salmonella* Heidelberg isolates recovered from ground turkey in 2006 were resistant to ceftiofur (*13*). CIPARS does not conduct ongoing surveillance of retail turkey, and we cannot ignore the possibility that retail turkey could be a source of ceftiofur-resistant *Salmonella* Heidelberg for humans as well. However, turkey consumption in Canada (4.7 kg per capita) was much lower than chicken consumption (33.2 kg per capita) in 2007 (*31*). Lastly, *Salmonella* Heidelberg has been reported in clinical samples from various other animal species in Canada (*12*), and exposure to sick animals could potentially be another source of infection. However, ceftiofur resistance in clinical *Salmonella* Heidelberg isolates remains anecdotal in species other than chicken and turkey (*12*).

Drug use monitoring in chicken is nonexistent in Canada. However, research data indicate a high level of ceftiofur use in Québec hatcheries in 2003–2004, where at least 78% of the lots surveyed in Québec abattoirs (M. Boulianne et al., unpub. data) had received ceftiofur in ovo. During that same period, ceftiofur resistance among retail chicken *Salmonella* Heidelberg isolates were >60%. The rapid and important 82% (*E. coli*) and 89% (*Salmonella* Heidelberg) declines in ceftiofur resistance in Québec retail chicken meat that followed in 2005–2006, as well as in Québec chicken *E. coli* and *Salmonella* isolates collected from passive surveillance of animal clinical isolates conducted by the Ministère de l’Agriculture, des Pêcheries et de l’Alimentation du Québec (MAPAQ) (*32*), is consistent with an effective voluntary withdrawal in 2005 and 2006. In 2007, the Québec broiler industry announced a potential partial reinstitution of ceftiofur use to control omphalitis in young chicks, with the intention of using the drug on a rotational basis and limiting its use to no more than 6 months per year (*32*). Again, CIPARS data from Québec retail chicken sampling in 2007–2008 demonstrating a reemergence of ceftiofur resistance among *E. coli* but at lower levels than in 2003–2004 are consistent with a partial return to ceftiofur use. The simultaneous reduction (and reemergence) in ceftiofur resistance in both retail chicken *E. coli* and *Salmonella* Heidelberg isolates and in clinical chicken *E. coli* and *Salmonella* isolates from MAPAQ surveillance support the hypothesis that the fluctuations in ceftiofur resistance most likely were driven by a common exposure (or reduction of exposure) to ceftiofur in chicken hatcheries, rather than simply being secondary to the natural spread and disappearance of a ceftiofur-resistant clone unrelated to ceftiofur use.

Although Ontario hatcheries had never announced an official withdrawal of ceftiofur use, a drop in ceftiofur resistance also was observed among chicken *Salmonella* Heidelberg isolates in Ontario in 2005. Although some argue that this proves the absence of an association between ceftiofur use and ceftiofur resistance in broiler chicken, movement of hatching eggs, broiler chicks (mostly from Québec to Ontario), and retail chicken meat between these 2 provinces could explain some of the similarities among *Salmonella* Heidelberg isolates in Ontario and Québec (*33*). The withdrawal in Québec might also have led Ontario broiler chicken hatcheries to temporarily decrease their use of ceftiofur in 2005.

In the absence of reliable comprehensive drug use information in the broiler chicken industry, we use resistance in commensal *E. coli* as a surrogate measure of the level of drug use (*34*). The high prevalence of ceftiofur resistance among *E. coli* isolates from British Columbia (almost half of the isolates in 2008 in that province), the increasing prevalence of resistance measured in Saskatchewan, and the 22% ceftiofur resistance among chicken *E. coli* isolates from Ontario when ceftiofur resistance prevalence was at its lowest level in Québec in 2006, indicates that ceftiofur use is unlikely to be restricted to the province of Québec. Lastly, in ovo ceftiofur use has also been reported in US chicken hatcheries (*35*).

Coselection of resistance to cephalosporins by exposure to other antimicrobials or to chemicals in the agricultural environment has been suggested as a confounding factor for the increase in observed resistance. Giles et al. (*36*) report the presence of the *sug*E gene on the same element as the *bla*_CMY-2_ gene in *Salmonella,* but the capacity of this gene to effectively confer resistance to quaternary ammonium compounds and provide coselection remains uncertain.

The levels of contamination of retail chicken with ceftiofur-resistant *E. coli* represent an additional concern. No selective media for ceftiofur-resistant strains was used, and the level of contamination of retail chicken with ceftiofur-resistant *E. coli* (and *Salmonella* Heidelberg) strains was most likely underestimated. Although this study describes exposure to commensal *E. coli*, such strains occasionally may cause infections in predisposed humans. In addition, the species *E. coli* includes a variety of strains commonly pathogenic for humans, and some strains from the normal flora of animals may carry a variety of virulence determinants that increase their potential for causing disease in humans (*37*). Poppe et al. (*38*) also demonstrated experimentally the acquisition of resistance to extended-spectrum cephalosporins by *Salmonella* serovar Newport from *E. coli* strains by conjugation in poultry intestinal tracts. In addition, molecular characterization of plasmids from field isolates demonstrates that identical *bla*_CMY-2_ plasmids can be found in both *Salmonella* and *E. coli* from the same chicken (P. Boerlin et al., unpub. data). Because the *bla*_CMY-2_ gene is horizontally transferable and is frequently observed in ceftiofur-resistant isolates of chicken origin, chicken could potentially be a reservoir of this gene for human pathogens, including *Salmonella* and others.

Except for anecdotal information, little information is available about drugs used by broiler chicken hatcheries and growers in Canada. The absence of on-farm drug use monitoring data prevents us from fully determining the effect of subtle changes in the level of use of ceftiofur (or other drugs) on resistance among bacteria recovered from chickens in Canada. Surveillance data from turkey or other nonsurveyed commodities would be useful to adequately quantify the contribution of each commodity to the overall number of cases related to ceftiofur-resistant *Salmonella* Heidelberg in humans. The impact of disinfectants used by the broiler industry at the farm or processing level on the selection of ceftiofur-resistant strains also needs to be assessed. Lastly, CIPARS is planning a burden-of-illness study to measure the impact of extended-spectrum cephalosporin resistance in *Salmonella* Heidelberg on human health.

Efforts undertaken by Québec chicken hatcheries to voluntarily withdraw use of ceftiofur in 2005–2006 coincided with a markedly reduced prevalence of ceftiofur-resistant *Salmonella* Heidelberg in retail chicken. This drop also effectively reduced the number of ceftiofur-resistant *Salmonella* Heidelberg infections in humans in this province during the same period. This reduction suggests that control of resistance to extended-spectrum cephalosporins is possible by managing ceftiofur use at the hatchery level. The partial reintroduction of ceftiofur use in Québec chicken hatcheries in 2007 with increasing rates of ceftiofur resistance after reintroduction, and indications that ceftiofur is used for the same purpose in other Canadian provinces, is of high concern. An increasing level of exposure to *E. coli* strains carrying horizontally transferable genes conferring resistance to extended-cephalosporins complicates the situation. To ensure the continued effectiveness of extended-spectrum cephalosporins to treat serious human infections, multidisciplinary efforts are needed to scrutinize, and where appropriate, limit use of ceftiofur in Canadian food animal production, particularly in chicken.
